# Subcutaneous hematoma of the lower abdomen and perineum following transperineal prostate biopsy: a case report and literature review

**DOI:** 10.3389/fonc.2026.1776163

**Published:** 2026-04-10

**Authors:** Zhiyong Liu, Jianhe Wu, Yuanwei Li, Qiang Lu, Yongjun Yang

**Affiliations:** 1 Department of Urology, Hunan Provincial People’s Hospital, The First Affiliated Hospital of Hunan Normal University, Changsha, Hunan, China; 2 Clinical Medical College, Hunan Normal University, Changsha, Hunan, China

**Keywords:** case report, lower abdomen and perineum, prostate cancer, subcutaneous hematoma, targeted transperineal biopsy

## Abstract

Prostate cancer (PCa) is the most common malignant neoplasm of the urogenital system, with definitive diagnosis currently relying exclusively on prostate biopsy. Although the transperineal approach is associated with a lower incidence of perioperative complications, postoperative subcutaneous hematoma in the perineal region may be overlooked due to its anatomically concealed presentation, leading to delayed clinical attention. This report presents a case of extensive subcutaneous hematoma involving the lower abdomen and perineum following transperineal prostate biopsy in a patient diagnosed with PCa. Prompt management with prostatic artery angiography and superselective prostatic artery embolization, supplemented by endocrine therapy, resulted in rapid resolution of the hematoma. At three-month follow-up, tumor biomarkers remained stable, and key biochemical parameters had returned to pre-biopsy baseline levels, indicating favorable disease control and recovery.

## Introduction

Prostate cancer (PCa) is the most common malignant tumor of the male urogenital system (1). According to the latest cancer statistics in China, an estimated 134,200 new cases and 47,500 deaths were projected for 2022 (2). Currently, prostate biopsy remains the gold standard for definitive diagnosis of PCa, with two primary approaches: transperineal and transrectal prostate biopsy (TRPB) (3). The transrectal approach is associated with a higher risk of perioperative infectious complications, including sepsis, bacteremia, and prostatitis, necessitating routine prophylactic antibiotic use; non-infectious complications include hematuria and rectal bleeding (4). In contrast, the transperineal approach is primarily linked to hematuria and urinary retention, with significantly lower infection rates (5). Due to growing concerns over antimicrobial resistance and the inherent infection risks of the transrectal route, current clinical guidelines increasingly recommend the transperineal approach as the preferred method for prostate biopsy (4, 6).

## Case description

**Figure 1:**
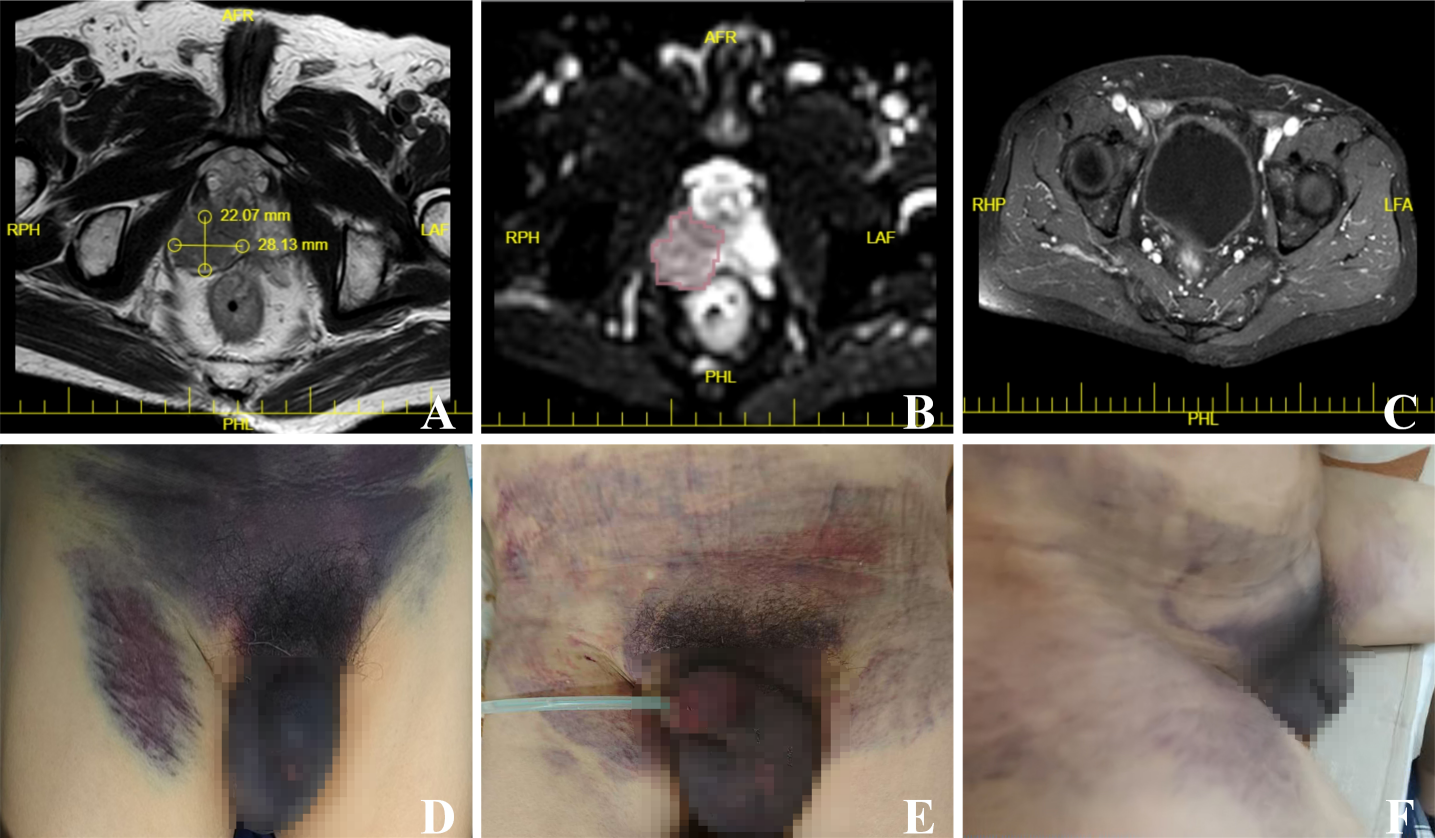
Radiologic findings and subcutaneous hematoma. **(A)** Axial T2-weighted MRI scan shows a solid lesion with slightly decreased signal intensity in the right lobe of the prostate. **(B)** The ADC value of the solid lesion in the right lobe of the prostate was significantly decreased. **(C)** No significantly enlarged lymph nodes were observed in the pelvic cavity, and there were no signs of involvement of adjacent organs. **(D)** Six days after TPPB, the patient developed extensive subcutaneous hematoma in the lower abdomen and perineum. **(E)** Ten days after TPPB, the subcutaneous hematoma showed initial signs of gradual absorption. **(F)** Twelve days TPPB, the subcutaneous hematoma had been nearly completely absorbed. Abbreviations: ADC, apparent diffusion coefficient; TPPB, transperineal prostate biopsy.

**Figure 2:**
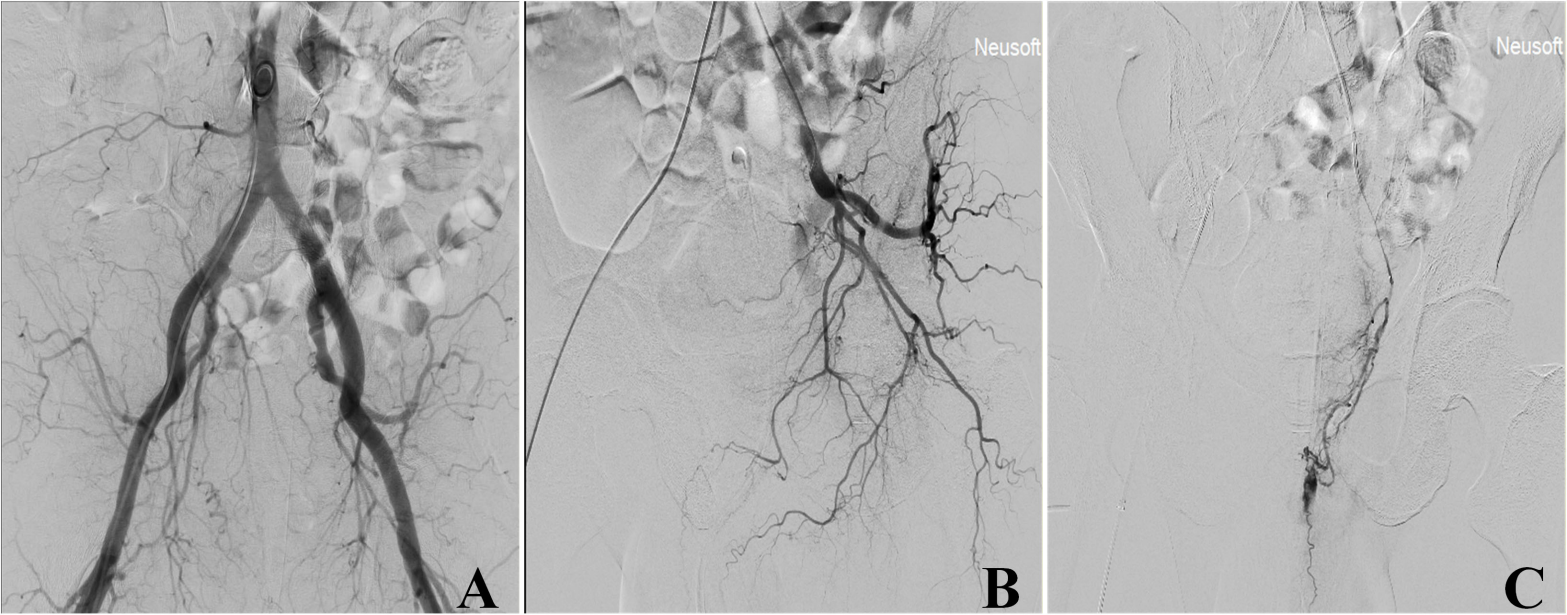
Pelvic angiography examination. **(A)** Angiography of the bilateral common iliac arteries and their branches. **(B)** Angiography of the left internal iliac artery and its branches. **(C)** Angiography of the left prostatic artery revealed contrast agent extravasation, prompting immediate superselective interventional embolization to achieve hemostasis.

This study presents a case of a 75-year-old male who developed lower abdominal and perineal hematoma following transperineal prostate biopsy (TPPB). The patient was referred to our hospital due to an elevated prostate-specific antigen (PSA) level persisting for one month. Upon admission, he reported no urinary symptoms—including dysuria, frequency, urgency, or hematuria—and denied systemic symptoms such as weight loss. Digital rectal examination revealed an asymmetrically enlarged prostate with a hard, irregular nodule in the right lobe. The central groove was obliterated, and the gland exhibited a non-tender consistency. The serum total PSA level was markedly elevated at 27.68 ng/mL, while all other laboratory parameters remained within their respective normal reference ranges. Multi-parametric magnetic resonance imaging (mpMRI) revealed a 28.13×22.07 mm nodule in the right posterior peripheral zone, exhibiting a low signal on the apparent diffusion coefficient imaging sequence, consistent with restricted diffusion (**Figures 1A, B**). Imaging finding further demonstrated the absence of significantly enlarged lymph nodes or evidence of adjacent organ involvement within the pelvic cavity (**Figure 1C**). Given that laboratory tests and imaging finding strongly indicated a PCa, the patient underwent mpMRI and transrectal ultrasound (TRUS) multi-modal image fusion-guided targeted TPPB. On day 6 post-biopsy, the patient was readmitted to the emergency department due to extensive subcutaneous hematoma involving the lower abdominal and perineal regions (**Figure 1D**). Upon admission, the patient presented with dysuria, urinary frequency, urgency, and gross hematuria. A urinary catheter was promptly inserted, and urgent laboratory evaluations were completed. These revealed abnormalities in coagulation parameters, complete blood count, and renal function, along with evidence of malnutrition. Given the patient’s extensive subcutaneous hematoma and significant decrease in hemoglobin levels within a short period of time (from 144 g/L to 55 g/L), active arterial bleeding was highly suspected clinically. Emergency pelvic angiography was therefore performed, which demonstrated unequivocal contrast extravasation from both prostatic arteries. Prompt superselective embolization of both prostatic arteries was subsequently performed using gelatin sponge particles (**Figures 2A-C**). Concurrently, the tissue specimen obtained through TPPB in the early stage was diagnosed as prostatic adenocarcinoma with a Gleason score of 8 (4 + 4), classified as grade 4 according to the WHO/ISUP grading criteria. Based on this, we initiated a novel combined endocrine therapy regimen, which involved the addition of rezvilutamide to androgen deprivation therapy (ADT). A follow-up examination on the 10th day after TPPB showed that the extensive subcutaneous hematoma began to absorb; by the 12th day, the hematoma had almost completely resolved (**Figures 1E, F**).

**Figure 3:**
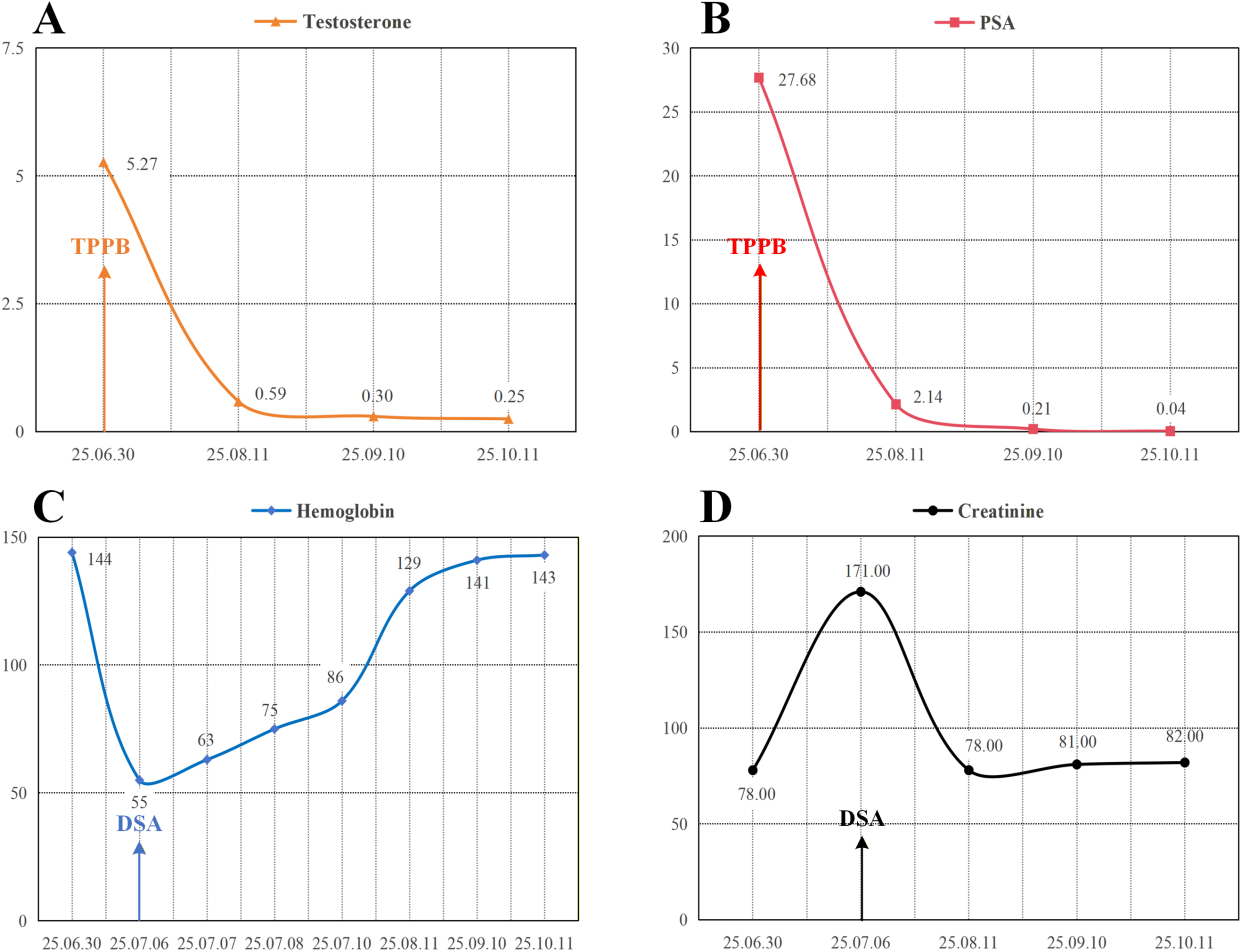
Laboratory test results obtained during the three-month follow-up period. **(A)** The serum testosterone level has decreased to within the castration-defined range. **(B)** The PSA level decreased to 0.04 ng/mL, representing a reduction of more than 99% from baseline. **(C)** The hemoglobin level has been restored to the preoperative baseline value. **(D)** The renal function test results have reverted to the preoperative baseline levels. Abbreviations: PSA, prostate-specific antigen; TPPB, transperineal prostate biopsy; DSA, digital subtraction angiography.

The indwelling catheter was removed on the seventh day following insertion, after which the patient did not report significant dysuria, urinary frequency, or gross hematuria. Following an uneventful 24-hour observation period, the patient was discharged without complications. Following initiation of medical castration therapy, standardized long-term follow-up is mandated by major international and national clinical practice guidelines for optimal disease monitoring and treatment safety. During the initial phase of treatment (the first 6 months), serum PSA levels should be monitored monthly. Once the condition stabilizes, follow-up can be extended to every 3–6 months for PSA, testosterone concentration, and liver and kidney function tests (7, 8). This case involves a newly diagnosed patient recently admitted to our center, who has completed a regular follow-up of 3 months to date. Serial measurements demonstrate persistent suppression of serum testosterone to castrate level (<50 ng/dL), and PSA has declined by 99.2% from baseline to 0.04 ng/mL (**Figures 3A, B**). Additionally, both hemoglobin and serum creatinine have returned to normal reference ranges, indicating that anemia and renal dysfunction have been corrected (**Figures 3C, D**).

## Discussion

Prostate biopsy remains the gold standard for the diagnosis of PCa. The overall incidence of perioperative complications is low, and most of them are self-limited. Based on etiological characteristics, perioperative complications can be divided into two major categories: infectious and non-infectious (9). Non-infectious complications include urinary retention, hematuria, hematospermia, biopsy-site bleeding, and erectile dysfunction. Among them, biopsy-site bleeding is most commonly observed in the rectoprostatic space. Studies have shown that after TRUS-guided TRPB, 14.5% of patients exhibited signs of rectoprostatic hematoma on imaging (10); in mpMRI-manual-guided TPPB, the incidence of asymptomatic rectoprostatic hematoma was 16%; whereas in mpMRI-guided robotic-assisted TPPB, this proportion rose to 30% (11). The vast majority of rectoprostatic hematomas are asymptomatic and can spontaneously absorb after symptomatic supportive and conservative treatment, such as bed rest and avoidance of straining during bowel movements. Other rare biopsy-site bleeding include the Retzius space, retroperitoneal space, prostate parenchyma, prevesical space, and anterior rectal wall (12–16). Literature reports are mostly individual cases with extremely low incidence; their clinical manifestations mainly include sudden lower abdominal pain and urinary irritation symptoms. Confirmed risk factors for biopsy-site bleeding include an increased number of puncture cores, use of anticoagulant drugs, and high-grade PCa (17). For patients with involvement of these rare sites and presenting with hemodynamic instability or progressive hematoma enlargement, prompt surgical exploration and hematoma removal should be performed, or referral to the interventional radiology department to assess the feasibility of minimally invasive interventions such as vascular embolization; whereas for asymptomatic or minimally symptomatic patients, conservative management strategies are recommended, including symptom-directed analgesia, selective prophylactic antibiotics, close clinical observation, and judicious use of follow-up imaging based on clinical evolution (10). To the best of our knowledge, this study is the first to report a case of extensive perineal and lower abdominal subcutaneous hematoma following multi-modal image fusion-guided targeted TPPB, with imaging confirmation of active prostatic bleeding.

In this case, the patient underwent TPPB using a combined biopsy strategy comprising two targeted cores and twelve systematic cores, for a total of fourteen needle passes. Histopathological examination confirmed prostate acinar adenocarcinoma with a Gleason score of 8 (4 + 4), reflecting high-grade malignancy and aggressive clinical behavior. This type of tumor frequently exhibit pathological angiogenesis, characterized by microvascular with markedly attenuated walls, absent or discontinuous smooth muscle coverage, and fragmented basement membranes—features collectively contributing to pronounced vascular fragility (18). During TPPB, the mechanical trauma from multiple needle traversals predisposes to injury of these structurally compromised intratumoral vessels as well as adjacent periprostatic small vessels, thereby triggering intraoperative or early postoperative hemorrhage. To compound this, the inherent vasoconstrictive insufficiency of such abnormal vasculature, coupled with impaired local platelet adhesion and fibrin deposition, severely limits spontaneous hemostasis. In cases of substantial hemorrhage, extravasated blood may breach the deep fascia barrier of the perineum and spread along the loose connective tissue spaces towards the lower abdominal wall. Especially, this extension is facilitated by the continuous fascial plane between the superficial perineal fascia (Colles fascia) and the membranous layer of the superficial abdominal fascia (Scarpa fascia), which merge inferior to the pubic symphysis. Furthermore, postoperative laboratory evaluation revealed coagulopathy—including prolonged prothrombin time (PT), reduced fibrinogen (FIB), and hypoalbuminemia—indicative of both impaired coagulation cascade function and compromised wound healing capacity. Thus, the confluence of tumor-specific microvascular pathology, procedural mechanical insult, and systemic hemostatic and nutritional deficits synergistically drove the cephalad extension of the subcutaneous hematoma from the perineum into the lower abdomen regions. Given the patient’s extensive subcutaneous hematoma and rapid progressive decline in hemoglobin levels, bilateral prostatic artery angiography and superselective prostatic artery embolization were immediately performed to promptly control active bleeding, aiming to precisely block the blood supply vessels of the abnormally proliferating tumor within the prostatic gland. Gelatin sponge particles were selected as the embolic agent during the procedure due to its good biocompatibility, controllable resorption, ease of operation, and high cost-effectiveness. It is commonly used for acute bleeding control, wound-related bleeding, and protective embolization of organ function (19). Postoperatively, a novel endocrine therapy regimen combining ADT with rezvilutamide was initiated. As a new generation of androgen receptor inhibitor, rezvilutamide can efficiently penetrate the blood-prostate barrier and potently inhibit the androgen receptor signaling pathway, thereby doubly suppressing tumor growth and tumor-related angiogenesis. This helps reduce intratumoral vascular fragility, stabilize neovascular structures, and subsequently lower the risk of rebleeding (20). It is worth noting that initiating this novel endocrine therapy in the early stage of acute bleeding not only achieves systemic control of the tumor but also synergizes with bleeding control by improving tumor microvascular stability.

This case not only underscores the critical importance of comprehensive perioperative management in TPPB complications, but also reinforces the pivotal role of preoperative patient education and postoperative self-care. Subcutaneous hematoma in the perineal region is frequently overlooked due to its anatomically concealed location and atypical clinical presentation, often manifesting with minimal or nonspecific symptoms. The detailed documentation of this rare complication—extensive subcutaneous hematoma involving both the lower abdomen and perineum—significantly enriches the current clinical literature and offers valuable insights for establishing standardized diagnostic approaches and therapeutic strategies. Importantly, early recognition and prompt intervention of active intraprostatic hemorrhage are essential to prevent progressive extension of the hematoma. Given that the patient in this case underwent prostate artery embolization, it is crucial to monitor the blood perfusion status of reproductive organs such as the prostate, scrotum, and penis during long-term follow-up. At the same time, dynamic assessment of the recurrence risk of subcutaneous hematoma at the puncture site, as well as the treatment response and control of the primary disease, should be conducted.

This study has several limitations. First, as a single-center case series with a limited sample size, its findings have constrained external validity and may not be generalizable to broader or more heterogeneous patient populations. Second, the descriptive nature of case-based reporting—coupled with inherent constraints in data completeness and granularity—precluded systematic identification, quantification, or causal inference regarding potential risk factors for complications. Third, the relatively short follow-up duration limits assessment of long-term hemodynamic changes in prostate arterial perfusion after prostate artery embolization, and thus the temporal relationship between vascular remodeling and clinical outcomes remains uncharacterized.

## Conclusion

The incidence of perioperative complications associated with TPPB is generally low. Due to the concealed anatomical location of the perineum and the absence of typical clinical manifestations (such as significant pain or swelling) in early bleeding, biopsy-site bleeding is often overlooked by both patients and medical staff. Therefore, systematic preoperative patient education and structured postoperative follow-up with clear time nodes are crucial for early identification of subcutaneous hematomas in the perineal region. Timely diagnosis can initiate intervention measures, effectively curb hematoma progression, and reduce the risk of severe complications such as secondary infection and tissue necrosis.

## Data Availability

The original contributions presented in the study are included in the article/supplementary material. Further inquiries can be directed to the corresponding authors.
